# Assessment of occlusion with the T-Scan system in patients undergoing orthognathic surgery

**DOI:** 10.1038/s41598-017-05788-x

**Published:** 2017-07-13

**Authors:** Jimoh Olubanwo Agbaje, Elke Van de Casteele, Ahmed S. Salem, Dickson Anumendem, Eman Shaheen, Yi Sun, Constantinus Politis

**Affiliations:** 1 OMFS-IMPATH Research Group, Department of Imaging and Pathology, Faculty of Medicine, Catholic University Leuven, Leuven, Belgium; 2 Oral and Maxillofacial Surgery Department, Faculty of Dentistry, Mansoura University, Mansoura, Egypt; 3 Statistician with the Centre for Educational Effectiveness and Evaluation of the Catholic University of Leuven and Biostatistician at SGS Life Science Services – Clinical Research, Mechelen, Belgium; 4 Faculty of Medicine, Hasselt University, Diepenbeek, Belgium

## Abstract

Dental occlusion varies among individuals, and achieving correct physiological occlusion after osteotomy is essential for the complex functioning of the stomatognathic system. The T-Scan system records the centre of force, first contact, maximum bite force, and maximum intercuspation. The aim of the present study was to investigate the usefulness and consistency of T-Scan in assessing occlusion before and after orthognathic surgery. Occlusal information was evaluated for 30 healthy adults with normal occlusion and 40 patients undergoing orthognathic surgery. T-Scan had a high degree of reliability for consecutive measurements (Pearson correlation, r = 0.98). For most parameters, occlusal distribution was better after surgery than before surgery. More teeth contributed to occlusion at maximum intercuspation after surgery than before surgery (14 vs. 10). In addition, the difference in the posterior force distribution was reduced after surgery (17.6 ± 13.8 vs. 22.7 ± 21.4 before surgery), indicating better occlusal force distribution after surgery. The maximum percentage force on teeth (p = 0.004) and the number of teeth contributing to occlusion (p < 0.001) also differed significantly. Thus, T-Scan is good for assessing occlusal discrepancies and can be used to portray the pre- and post-operative occlusal contact distribution during treatment planning and follow-up.

## Introduction

The masticatory system is made up of the teeth, periodontal tissues, muscles of mastication, and temporomandibular joint (TMJ). Physiological restoration of occlusion is important for a healthy functioning masticatory system^1–3^. Dental occlusion varies among individuals according to the size, shape, and position of the teeth; timing and sequence of eruption; dental arch size; and shape and pattern of craniofacial growth^4^. The position of the teeth within the jaw and the mode of occlusion are determined by developmental processes that affect the teeth and their associated structures during the period of formation, growth, and post-natal modification^1–3^. Any anomaly occurring during or after developmental processes can result in maxillary and/or mandibular deformities^5, 6^. Orthognathic procedures to treat maxillary and/or mandibular deformities result in better masticatory function, reduced TMJ pain, and improved facial aesthetics^7–9^. Achieving correct physiological occlusion after osteotomy is essential for the complex functioning of the stomatognathic system. Occlusion can be defined as contacts between teeth that are “static” when the mandible is closed and stationary or “dynamic” when the mandible is moving relative to the maxilla^4^.

**Figure 1:**
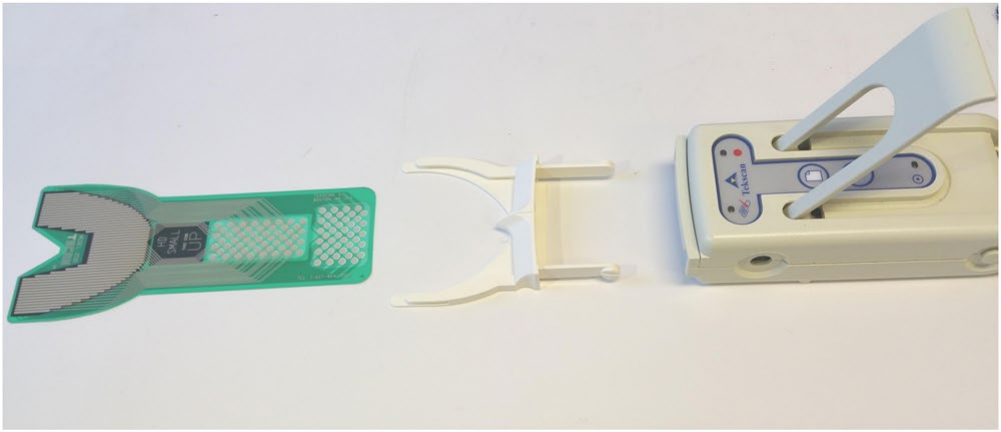
T-Scan system (T-Scan III, Software version 8.0.1, Tekscan, Inc., Boston, MA, USA).

**Figure 2:**
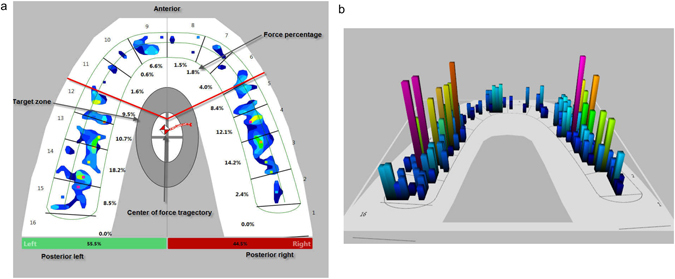
Occlusal force distribution using T-Scan III, Software version 8.0.1. (**a**) Two-dimensional and (**b**) three-dimensional images of a healthy participant.

T-Scan (T-Scan III, Software version 8.0.1, Tekscan, Inc., Boston, MA, USA) is a digital occlusion analysis system that records and measures tooth contact, force, and timing in real-time using a thin, flexible, pressure-sensitive bite transducer embedded in a dental arch-shaped recording sensor^4, 10–12^ (Fig. 1). The occlusal data obtained from T-Scan can be displayed graphically for analysis in two or three dimensions (Fig. 2) or as a dynamic movie that can be analysed stepwise. The occlusal force distribution, occlusal interference, and relative force of each interference can be determined from the recorded occlusal data. The T-Scan records patient parameters, such as the centre of force, demonstrating the symmetry of the occlusal force; first contact, the area of early contact between maxillary and mandibular teeth; maximum bite force; and maximum intercuspation, the occlusal position of the mandible in which the cusps of the teeth of one arch fully interpose themselves with the cusps of the teeth of the opposing arch. Maximum intercuspation is an important jaw position that defines both the anterior-posterior and lateral relationships of the mandible and maxilla, as well as the superior-inferior relationship known as the vertical dimension of occlusion. Maximum intercuspation is an important consideration when evaluating an orthodontic patient.

Masticatory performance has been correlated with occlusion and the occlusal contact area; patients with better masticatory performance have well-distributed occlusal contact areas, whereas patients with malocclusion have lower masticatory performance than those with normal occlusion^13, 14^. Studies have also shown that impaired masticatory function can adversely affect the quality of life^8, 9^.

The aim of the present study was to investigate the usefulness of T-Scan in assessing a patient’s occlusal condition before and after orthognathic surgery by assessing the force distribution on the patient’s arch before and after surgery, the number of teeth in the arch that contribute to the patient’s occlusion at maximum intercuspation before and 1 year after orthognathic surgery, the location of the centre of force to determine the symmetry of the force distribution in the arch, and the consistency of T-Scan measurements during follow-up intervals. In addition, this study explored the different patterns of T-Scan images observed in healthy participants and orthognathic surgery patients in an attempt to group them for diagnostic purposes.

## Materials and Methods

### Study sample

The study sample included a control group and an orthognathic treatment group. The control group included 30 healthy adults (16 women and 14 men >18 years of age, mean age ± SD 30.8 ± 9.7 years) with normal occlusion (angle class I with a normal line of occlusion without malposed or rotated teeth) recruited through the maxillofacial unit of the university hospital. Additional inclusion criteria were full dentition (32 teeth) and no history of previous orthodontic treatment. Exclusion criteria were the presence of TMJ problems that limit mouth opening and malocclusion, such as open bite, increased overjet or reverse overjet, cross bite, and skeletal anomalies with occlusal disturbance. The orthognathic treatment group included 40 patients (25 women, 15 men, mean age ± SD 26.2 ± 10.1 years) scheduled for orthognathic surgery at Leuven University Hospital, Department of Oral and Maxillofacial Surgery. This group included patients with skeletal anomalies with occlusal disturbance, increased or reverse overjet, open bite, or prognathic or retrognathic jaws (Table 1). Patients with cleft palate, syndromic patients, and patients who underwent previous orthognathic surgery were excluded. The study was performed in accordance with Helsinki Declaration II. The study protocol was approved by the ethics committee of UZ Leuven S55873. All procedures were fully explained to all participants, who provided written informed consent.

**Table 1:** Malocclusion and surgical characteristics of the orthognathic surgery patients included in the study.

Orthognathic surgery patients	
	Number of patients (%)
Angle classification of malocclusion:	
Class I	14 (35%)
Class II	20 (50%)
Class III	6 (15%)
Maxillary and mandibular incisor relationship: (mean ± SD)	
Open bite (2.1 mm ± 1.37 mm)	10 (25%)
Overjet (4.75 mm ± 2.74 mm)	24 (60%)
Reverse overjet (3.0 mm ± 2.08 mm)	6 (15%)
Surgery type:	
Le fort I	5 (12.5%)
BSSO	18 (45%)
Bimaxillary surgery	17 (42.5%)

### T-Scan occlusion recording

Occlusal information was obtained for both groups using the T-Scan device (T-Scan III, Software version 8.0.1). In the control group, T-Scan recordings were collected once, but in orthognathic surgery patients a recording was obtained before the operation and at each follow-up (6 weeks, 3 months, 6 months, and 1 year). Multi-bite T-Scan (Fig. 1) recordings were made with the participant sitting upright in the dental chair. The sensor sends real-time occlusal contact and force information to the accompanying Windows-based software package. This information is displayed in two and three dimensions as a continuous force “movie” of the entire recorded occlusal contact event. In addition, four repeated occlusal recordings were performed in two participants at weekly intervals to assess the consistency of the T-Scan measurement during follow-up. The occlusal data of the control group and the pre-operative and 1-year post-operative data of the orthognathic surgery patients were transferred into a spreadsheet for further analysis.

### Occlusion analysis

The following measurements were obtained from the occlusal data: force distribution in the patient’s arch (Fig. 2), number of teeth that contribute to the occlusion at maximum intercuspation, location of the centre of force in the arch to determine the symmetry of the force distribution in the arch. In order to analyse the T-Scan patterns, the occlusal force information of the arch at maximum intercuspation was grouped into three regions (Fig. 2): anterior (canine to canine, numbers 6–11), posterior right (premolar to molars, numbers 1–5), and posterior left (premolar to molars, numbers 12–16). The force distribution in all three regions was assessed in all participants, as well as the difference in percentage force distribution between the posterior right and left, the number of teeth that contribute to occlusion, and the maximum percentage force on the tooth at maximum intercuspation.

### Statistical analysis

The ‘patients’ characteristics are presented using descriptive statistics. Counts and percentages were used to summarize categorical variables. The mean, standard deviation, and range were calculated for the percentage occlusal force for the three regions. The absolute difference in percentage force between the posterior right and left, number of teeth that contribute to occlusion, and maximum percentage force on teeth for patients (pre- and post-surgery) and controls were calculated.

The statistical analysis was carried out using SAS version 9.4 (SAS Institute, Inc., Cary, NC, USA). The Wilcoxon signed-rank test was applied to assess significant differences between pre- and post-operative data. Non-parametric ANOVA was used to assess the variability within and between groups for the chosen measurement (anterior percentage force, posterior right and left percentage forces, difference between posterior percentage forces, number of teeth that contribute to occlusion, and maximum percentage force on teeth). P < 0.05 was considered significant. The consistency of occlusal information obtained from the T-Scan at different time intervals was tested on four repeated measurements in two participants. Cronbach’s alpha coefficient of reliability was used. The reliability was estimated by correlating the repeated measurements after administering the test two or more times (up to four times). Pearson r is the index of correlation most often used in this context.

## Results

### T-Scan reproducibility

The Cronbach’s alpha coefficient (estimated by Pearson correlation coefficients) of the overall forces on the arch was 0.98. For different regions of the occlusal arch, the alpha coefficient ranged from 0.92 (anterior) to 0.98 (posterior left). The Pearson r (index of correlation) ≥0.7 shows that the T-Scan occlusal reading was consistent, and the value is above which a measuring tool expresses strong reliability^15^.

### Percentage force distribution

The percentage force distributions in the anterior and posterior regions of the control and treatment group are shown in Table 2. The mean percentage anterior force was 14.3% ± 5.8%, and the mean percentage posterior forces were 50.1% ± 5.7% on the right and 43.9% ± 4.9% on the left. Between 12 and 16 teeth contributed to occlusion at maximum intercuspation. In 28 of the 30 controls, the centre of the force was in the “target zone” depicted in the T-Scan software (white/grey ellipse area in the middle of the arch, Fig. 2a), indicating occlusal force symmetry. The maximum percentage occlusal force recorded on any tooth was 18.6% ± 3.7%.

**Table 2:** T-Scan analysis parameters of the control group and the treatment group before surgery and 1 year after.

	Control		Pre-operative		Post-operative	
	Mean ± SD (%)	Range (%)	Mean ± SD (%)	Range (%)	Mean ± SD (%)	Range (%)
Anterior region	14.3 ± 5.8	1–24.1	24.5 ± 29.3	0–100	21.6 ± 14.5	1.7–62.3
Posterior right	50.1 ± 5.7	34.2–58.7	39.1 ± 21.8	0–91.5	40.6 ± 15.2	6.1–81.9
Posterior left	43.9 ± 4.9	33–55.8	38.71 ± 20.3	0–75	39.3 ± 11.2	12.6–62.2
Difference posterior left and right	9.7 ± 5.2	1–19.3	22.7 ± 21.4	0–90.6	17.6 ± 13.8	0.1–65.8
Teeth in occlusion	15 ± 1.3	12–16	10.2 ± 3.44	3–16	13.5 ± 2.6*	3–16
Maximum concentrated force	18.6 ± 3.7	13.8–29.6	29.5 ± 11.4	11.4–56.6	22.9 ± 7.4*	11.8–49.9

The asterix (*) indicates the statistical significant differences found in the treatment group between the two time points using a Wilcoxon signed-rank test with p < 0.05.

**Figure 3:**
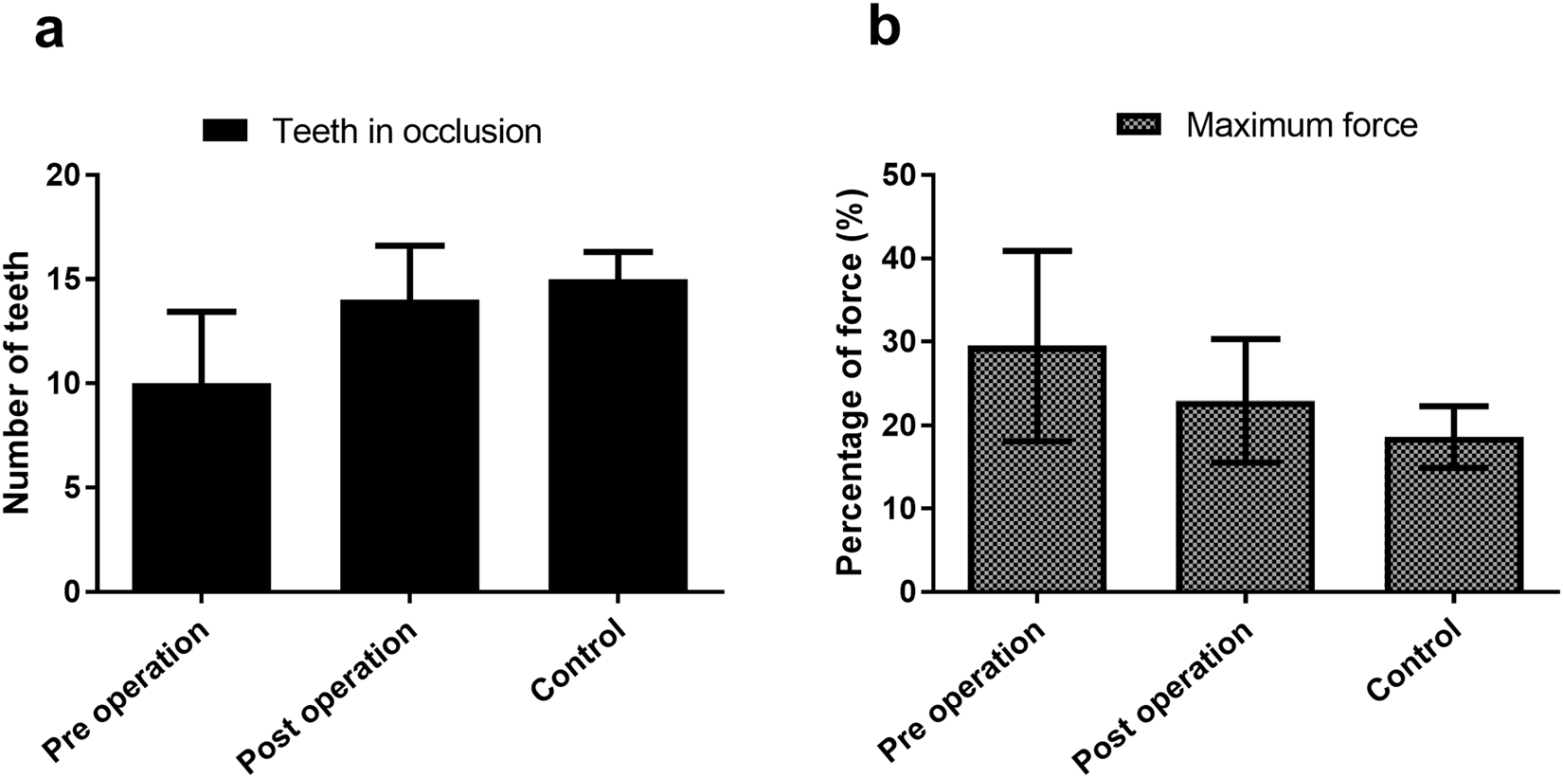
Comparison of (**a**) the number of teeth in occlusion and (**b**) maximum percentage force on teeth.

A summary of the T-Scan occlusal information obtained for the orthognathic surgery patients before and after surgery is presented in Table 2. For most of the parameters, the post-operative information indicated a better occlusal distribution outcome than before the operation. More teeth contributed to occlusion at maximum intercuspation after surgery (13.5 ± 2.6 teeth) than before surgery (10.2 ± 3.44 teeth, Fig. 3) which is closer to the control group outcome (15 ± 1.3 teeth). The difference in the posterior percentage occlusal force distribution was also reduced after surgery (17.6% ± 13.8%) compared to before surgery (22.7% ± 21.4%); thus, there was better occlusal distribution after surgery. The maximum percentage occlusal force on a tooth before and after surgery was 29.5% ± 11.4% and 22.9% ± 7.4%, respectively. No significant difference was found between the anterior (p = 0.742), posterior left and right percentage forces (p = 0.787 and p = 0.843), or between posterior percentage forces (p = 0.091) for the pre- and post-operative measurements. However, in the Wilcoxon signed-rank test, a significant difference was found for the maximum percentage force on teeth (p = 0.004) and the number of teeth contributing to occlusion (p < 0.001).

### Occlusal pattern

**Figure 4:**
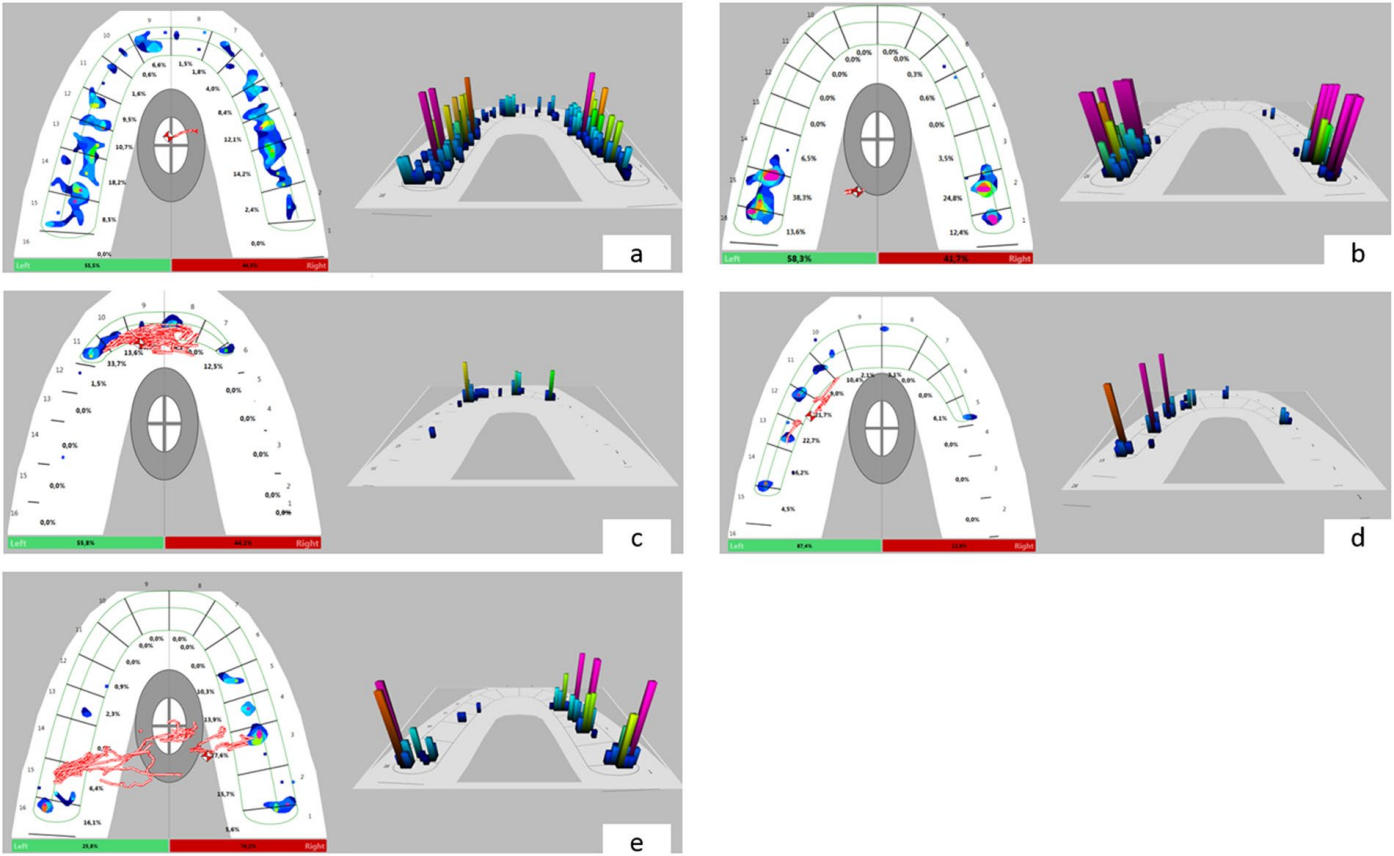
Patterns of occlusion before and after surgery using T-Scan III, Software version 8.0.1. (**a**) Normal occlusion, (**b**) anterior open bite, (**c**) bilateral posterior open bite, (**d**) unilateral posterior open bite right, (**e**) anterior and unilateral posterior open bite left.

Similar three-dimensional T-Scan images in participants (control and orthognathic surgery patients) at maximum intercuspation were grouped together. From this grouping, five patterns emerged based on the absence of contact (further defined as “open bite”), which was striking and meaningful. The observed patterns are presented in Table 3 and Fig. 4. In addition, correlations between the angle classifications of malocclusion among orthognathic surgery patients and the five T-Scan occlusal patterns were evaluated.

**Table 3:** T-Scan occlusal patterns in pre-surgical patients.

Group	Patterns
1	Normal occlusion
2	Anterior open bite
3	Bilateral posterior open bite
4	Unilateral posterior open bite (right or left)
5	Anterior and unilateral posterior open bite (right or left)

The T-Scan occlusal pattern did not correlate with the angle classification of malocclusion. A similar occlusal pattern could be present in, for example, angle class II or class III malocclusions depending on the position of the teeth on the arch and relative to the opposing jaw.

## Discussion

**Figure 5:**
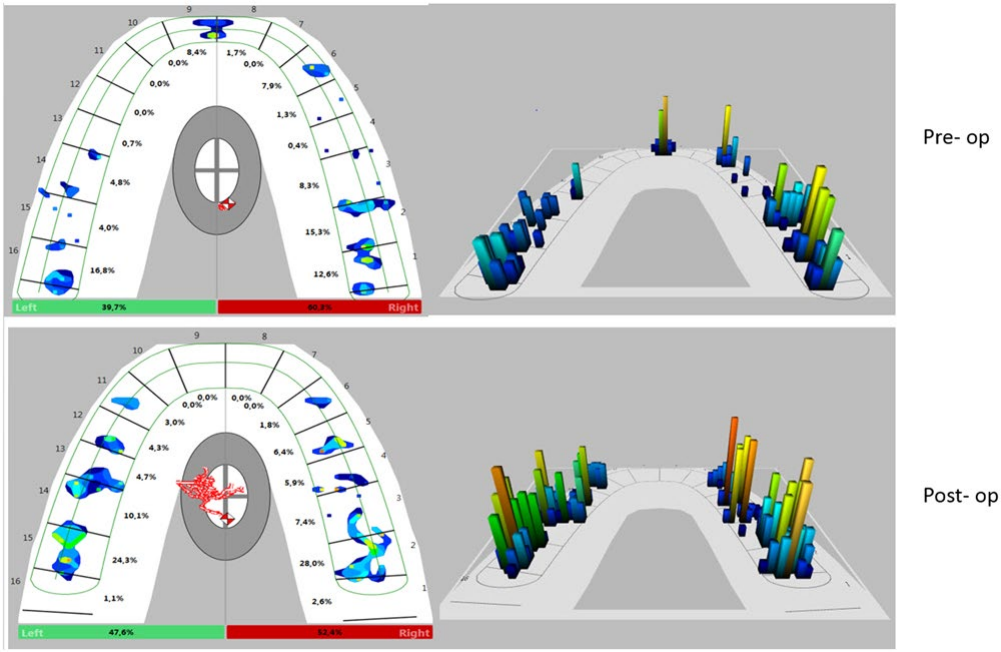
Occlusal force distribution before and 1 year after surgery using T-Scan III, Software version 8.0.1.

The T-Scan system has numerous clinical applications in dentistry and oral and maxillofacial surgery^3, 10, 16–20^. Occlusal adjustment is usually performed following orthognathic surgery, implant procedures, or dental prostheses, and these adjustments can be guided precisely by T-Scan analysis. Bite testing using T-Scan allows the evaluation of occlusal contacts prior to making a bite adjustment. The T-Scan can indicate premature contact and the load distribution on teeth, and provide measurable force and time information that ensures proper occlusal adjustment. In implant dentistry, the discovery of premature contacts allows early intervention to prevent future problems. In orthodontics and orthognathic surgery, the absence of contacts or the presence of asymmetric contacts can indicate the need for further correction and finishing. A T-Scan is much more informative than an examination with articulator paper due to simultaneous examination of the entire arch with proper visualization of the problem. Information from the T-Scan system helps locate and identify traumatic occlusal contacts and can be used to compare occlusal force symmetry before and after treatment (Fig. 5). In addition, the T-Scan system can be used to evaluate changes in the state of a patient’s occlusion after orthognathic surgery and be a useful tool for patient follow-up and the diagnosis or evaluation of relapse after treatment.

The control group presented with the features of balanced occlusion, such as posterior balance force, centre of force in the target area, and most teeth contributing to occlusion. A significantly higher occlusal force percentage was found in the posterior region than the anterior region, which is in agreement with previous studies^21–23^. In addition, a slight difference in the bilateral force distribution was found, indicating slight asymmetry, even in patients with normal occlusion. Though the occlusion is habitual, as the closure is not controlled/guided, the mean difference between posterior percentage forces was minimal (<10%). In addition, the centre of force remained in the “target zone” (white/grey ellipse area in the middle of the arch) in 93% (28/30) of participants. A minimal difference was found between the right and left posterior percentage force, as well as the location of the centre of the trajectory point to occlusal symmetry in the control group. Furthermore, better load distribution was observed with an average of 15 teeth in occlusion at maximum intercuspation. The controls presented with a range of percentage forces in the anterior and posterior regions, possibly due to differences in the patients’ build and arch shapes^4^.

Before surgery in the orthognathic surgery patients, the mean difference between posterior percentage forces was >20%, with an average of 10 teeth contributing to occlusion and a high maximum percentage force (as high as 56%) on a tooth. We observed that subjects with malocclusion had fewer teeth contributing to occlusion than subjects with normal occlusion, a finding that is consistent with previous studies^24, 25^. Better force distribution was measured 1 year after surgery compared to the pre-operative condition; more teeth contributed to occlusion post-operatively than pre-operatively. The difference in the percentage force between the posterior left and right arch was also reduced, and the maximum percentage force was reduced. These findings are indicative of a better occlusal force distribution after surgery than before surgery (Fig. 5). Good occlusion with symmetric occlusal contacts can influence the stability of orthognathic treatment. The number, location, and size of occlusal contacts, as well as the forces applied, are important for good functioning of the TMJ system^26^. As absolute bite force was not measured, but rather the occlusal distribution of impacting forces on the arch, only a qualitative analysis of improvements in the stomatognathic system is realized using T-Scan. Despite the improvement in occlusal symmetry, the treated group was not as good post-operatively as the control group. Although surgery can improve occlusion, perfect occlusion may be difficult to attain and post-operative orthodontics remain necessary.

Dees *et al*.^27^ described the earlier version of T-Scan (T-Scan I) as a major technical innovation in functional diagnostics, and the same holds true for the version that came after. This diagnostic function will aid in patient planning and assist in post-operative follow-up. One important property of a diagnostic tool is reliability, which is the ability of the instrument to yield the same result when measured at different times under similar conditions^28^. We assessed the consistency of T-Scan to produce the same occlusal contact reading during the follow-up period by obtaining four repeated measurements from two participants. Our findings were in agreement with those of Cerna *et al*.^29^, who found that T-Scan has a high degree of reliability when used to perform consecutive measurements, and González *et al*.^30^, who found no significant differences between the number of contacts on each tooth after four bites performed in an maximum intercuspation position using T-Scan I. Other studies also demonstrated that the T-Scan system is a reliable method for analysing and evaluating occlusal contact distribution in maximum intercuspation^30–32^. The observed T-Scan patterns have no direct correlation with angle classification; any of the observed patterns can be seen in angle class I, II, or III.

Oral and maxillofacial surgeons classify occlusion using angle classification, which is based on the relationship between the first molars of the upper and lower jaw. T-Scan is a good tool for assessing the forces and bite dynamics, but it is not able to determine whether the force comes from the maxillary or mandibular teeth because it only records the force in-between the teeth. Thus, the T-Scan patterns do not correlate with the angle classification. However, T-Scan patterns provide complementary information that the surgeon cannot assess clinically.

The observed T-Scan pattern being found in patients after orthodontic alignment emphasizes the need for additional surgery after orthodontic treatment to achieve balanced occlusion. Kalachev *et al*.^33^ found T-Scan to be a valuable and reliable system for the localization and distribution of occlusal contacts in dynamic articulation. Although this study evaluated participant occlusion at maximum intercuspation, we think it is a good representation of a patient’s occlusion even during mastication. According to Iwase *et al*.^34^, the maximum occlusal contact area during chewing is nearly identical to the statically determined maximum possible occlusal contact area.

Therefore, T-scan is a way to assess how a patient’s bite is functioning. Further studies evaluating occlusion results may relate it to functional needs^35^.

## Conclusions

T-Scan technology provides comprehensive information about a patient’s occlusion and can help clinicians assess treatment outcomes. T-Scan is a good tool for the assessment of occlusal discrepancies and can be useful during both treatment planning and the follow-up period, especially in orthognathic surgery patients. The system may also allow relapse to be detected earlier.
